# Clinical and histopathological factors associated with Ki-67 expression in breast cancer patients

**DOI:** 10.3892/ol.2015.2852

**Published:** 2015-01-07

**Authors:** GUL ALCO, ATILLA BOZDOGAN, DERYA SELAMOGLU, KEZBAN NUR PILANCI, SITKI TUZLALI, CETIN ORDU, SEFIK IGDEM, SAIT OKKAN, MAKTAV DINCER, GOKHAN DEMIR, VAHIT OZMEN

**Affiliations:** 1 Department of Radiation Oncology, Gayrettepe Florence Nightingale Hospital, Gayrettepe, Istanbul 34349, Turkey; 2 Department of BiostatisticsSurgery, Istanbul Florence Nightingale Hospital, Gayrettepe, Istanbul 34349, Turkey; 3 Department of Breast Surgery, Istanbul Florence Nightingale Hospital, Gayrettepe, Istanbul 34349, Turkey; 4 Department of Medical Oncology, Istanbul Bilim University, Gayrettepe, Istanbul 34349, Turkey; 5 Department of Pathology, Istanbul Florence Nightingale Hospital, Gayrettepe, Istanbul 34349, Turkey; 6 Department of Radiation Oncology, Istanbul Bilim University, Gayrettepe, Istanbul 34349, Turkey; 7 Department of General Surgery, Istanbul Medical Faculty, Istanbul University, Capa, Istanbul 34390, Turkey

**Keywords:** breast cancer, Ki-67, cut-off value, prognostic factors, molecular subtypes, age

## Abstract

The aim of the present study was to identify the optimal Ki-67 cut-off value in breast cancer (BC) patients, and investigate the association of Ki-67 expression levels with other prognostic factors. Firstly, a retrospective search was performed to identify patients with stage I–III BC (n=462). A range of Ki-67 index values were then assigned to five groups (<10, 10–14, 15–19, 20–24 and ≥25%). The correlation between the Ki-67 index and other prognostic factors [age, tumor type, histological and nuclear grade, tumor size, multifocality, an *in situ* component, lymphovascular invasion (LVI), estrogen and progesterone receptor (ER/PR) expression, human epidermal growth factor receptor (HER-2) status, axillary involvement and tumor stage] were investigated in each group. The median Ki-67 value was revealed to be 20% (range, 1–95%). A young age (≤40 years old), tumor type, size and grade, LVI, ER/PR negativity and HER-2 positivity were revealed to be associated with the Ki-67 level. Furthermore, Ki-67 was demonstrated to be negatively correlated with ER/PR expression (P<0.001), but positively correlated with tumor size (P<0.001). The multivariate analysis revealed that a Ki-67 value of ≥15% was associated with the largest number of poor prognostic factors (P=0.036). In addition, a Ki-67 value of ≥15% was identified to be statistically significant in association with certain luminal subtypes. The rate of disease-free survival was higher in patients with luminal A subtype BC (P=0.036). Following the correlation analysis for the Ki-67 index and the other prognostic factors, a Ki-67 value of ≥15% was revealed to be the optimal cut-off level for BC patients.

## Introduction

Breast cancer (BC) is a heterogeneous disease, with several different subtypes identified by immunohistochemical analyses and genetic array testing (1). Multigene tests have revealed that tumor proliferation has a significant effect upon predicting the risk of disease recurrence (2,3). Although histopathological findings are used for disease management in patients with BC, the assessment of tumor proliferation, in addition to conventional parameters, has become a key factor for treatment decisions (4).

In addition to the thymidine-labeling index, a range of techniques are available to assess the rates of tumor cell proliferation. These include the calculation of mitotic figures in stained tissue segments, flow cytometric analysis to identify the proportion of cells in the S phase of the cell cycle and the expression analysis of the proliferating cell nuclear antigen or cyclins E and D (5–8).

The Ki-67 antigen is a labile, non-histone nuclear protein that was identified in the early 1980’s. Ki-67 regulates the cell cycle, is associated with cellular proliferation and is the most widely used proliferation marker (9). Previous studies have demonstrated that Ki-67 is expressed in all the active cell cycle phases, and not the resting G_0_ phase. Furthermore, Ki-67 has been used as a biomarker to assay the growth fraction of a given cell population (10,11). A study that analyzed samples of normal breast tissue revealed that that Ki-67 was expressed at low levels (<3% of cells) in estrogen receptor (ER)-negative cells, but was not detectable in ER-positive cells (12). Immunostaining techniques that use monoclonal Ki-67 antibodies are able to assess the growth fraction of neoplastic cell populations. Although Ki-67 is an accepted prognostic marker, the role of the protein in the management of BC is unclear. At present, a standard operating procedure, or generally accepted cut-off definition, are not defined for Ki-67 (13,14).

Despite the observation that high Ki-67 levels are associated with worse prognoses and survival rates in patients with early BC (8,15), the marker has not yet been implemented for routine clinical use. Due to insufficient quality assurance and existing data, The College of American Pathologists (CAP) has not advised the routine use of Ki-67 screening for the prognosis of patients with newly-identified BC (16). However, at the 2011 and 2013 St. Gallen Consensus Conferences, the use of Ki-67 screening was recommended for the analysis of cellular proliferation, and for identifying the differentiation status of luminal A and B tumors (1,17). This was in agreement with a study published by Perou *et al* (18), which presented notable results with regard to intrinsic molecular BC subtypes.

Despite a decrease in the mortality rates and the number of BC cases in developed countries, the incidence of BC in developing countries, such as Turkey, is increasing (19). In addition, the prognoses and survival rates of patients are worse in developing countries due to a higher incidence of poor prognostic factors [including triple-negative BC (TNBC), tumor grade and proliferative indexes, and low hormone receptor (HR) positivity statuses], diagnoses at advanced stages and insufficient treatment regimens. A previous study revealed that in the past two decades, the incidence of BC has increased by more than two times in Turkey, with 20% of patients <40 years old, and half premenopausal (19). Although the results of this study may be due, in part, to the particular age structure of the population in Turkey, age-adjusted analysis revealed that there was a higher proportion of young females who had presented with BC in Turkey. Within this analysis, invasive ductal carcinoma (IDC) was identified as the most common BC histology (82%), with the ER, progesterone receptor (PR) and human epidermal growth factor-2 (HER-2) receptor positive in 67, 52 and 23% of patients, respectively (19). Furthermore, >50% of the patients had a histological grade (HG) III-type tumor, a high tumor proliferation rate and lymphovascular invasion (LVI).

The current study aimed to investigate the role of Ki-67 as a prognostic marker, to identify any correlations between Ki-67 expression and other clinical and histopathological parameters, and to determine the optimal cut-off value of Ki-67 in a large cohort of females with BC.

## Materials and methods

### Patient selection, treatment and follow-up

Since January 2010, the Ki-67 index has been routinely used as a prognostic factor for patients with BC at the Breast Health Center, Florence Nightingale Hospital (Istanbul, Turkey). Between January 2010 and February 2013, 462 patients with invasive BC underwent surgery and their details were prospectively recorded. The electronic data from this cohort was then retrospectively analyzed. The follow-up period for this analysis continued until October 2013. The study was performed in accordance with the REMARK criteria (20). The patients included within the present study did not receive neoadjuvant treatment, and had a minimum one-year follow-up. Patients with a past malignancy, prior to developing BC, and patients with syncrone/metacrone bilateral BC were not included. Written informed consent was obtained from all patients and this study was approved by the ethics commitee of Istanbul Bilim University (Istanbul, Turkey).

Current international guidelines were applied for treatment selections in a multidisciplinary tumor board (17). The Ki-67 value was taken into consideration for the treatment decisions, together with all other clinical and histopathological risk factors. The patients received doxorubicin (60 mg/m^2^) and cyclophosphamide (600 mg/m^2^) every 3 weeks for four cycles, node positive patients also received paclitaxel (175 mg/m^2^ every 3 weeks for 4 doses) or (80 mg/m^2^ weekly for 12 doses) or, docetaxel (100 mg/m^2^ every 3 weeks for 4 doses or 35 mg/m^2^ weekly for 12 doses) and radiation therapy to the breast [median dose, 50 Gy (range, 46–50 Gy)], tumor bed [median dose, 14 Gy (range, 10–16 Gy)] and lymphatics [median dose, 46 Gy (range, 46–50 Gy)], when indicated. Hormone-naive patients were administered tamoxifen (20 mg, once a day) or aromatase inhibitors (1 mg anastrozole, once a day; 2.5 mg letrosole, once a day) for five years. In the absence of any medical contradictions, patients with HER-2 overexpression received trastuzumab for a one-year period. The patients were monitored every three months for the first two years, and every six months thereafter.

### Immunohistochemistry

The automatized immunohistochemical staining was performed using the Ventana Benchmark LT (Ventana Medical Systems, Tucson, AZ, USA) according to the manufacturer’s instructions. The monoclonal rabbit Ki-67 antibody (clone SP6; dilution 1:100; Biocare Medical, Concord, CA, USA), with an incubation time of 48 min, was used as the primary antibody. The Ki-67 immunostaining was performed by an experienced breast pathologist. The immunohistochemical analysis of Ki-67 was conducted following recommendations from the International Ki-67 in Breast Cancer Working Group (21). To calculate the Ki-67 proliferative index, the percentage of positively-stained cells, within the total number of malignant cells, were counted from the whole tissue sections. ‘Positive staining’ was regarded as the appearance of nuclear staining, of any intensity, within the tumor cells. The ‘hot spots’ at the edge of the invasive tumor were scored. The scoring was performed by counting a minimum of 500 invasive tumor cells. The normal ducts, lymphocytes and mitotic figures were used as internal positive controls, and sections from lymph nodes containing germinal centers were used as external positive controls.

The ER and PR expression analysis was performed using a rabbit monoclonal anti-human ER antibody (clone SP1; Neomarkers Inc., Fremont, CA, USA) at a 1/100 dilution, and a mouse monoclonal anti-human PR antibody (clone SP2; Neomarkers Inc.) at a 1/50 dilution. The ER and PR status was defined as the percentage of immunoreactive cells with an intranuclear staining of any intensity. The intranuclear staining of at least 1% of the cells was interpreted as a receptor-positive result (22). The HER-2 expression analysis was performed using a mouse monoclonal antibody (clone SP3; Neomarkers Inc.) at a 1/100 dilution. The interpretation analysis was performed according to the American Society of Clinical Oncology/CAP guidelines (23).

### Definition of molecular subtypes

The molecular subtypes were defined as luminal A or B (B1/B2), HER-2 enriched and triple-negative (Table I).

### Study design

In order to identify the optimal cut-off value of Ki-67, the distribution of Ki-67 immunostaining levels were divided into five groups (<10, 10–14, 15–19, 20–24 and ≥25%). The correlation between Ki-67 and other conventional prognostic factors [age, tumor type, multifocality/multicentricity, HG, according to the Nottingham grading system (24), nuclear grade (NG), according to Fisher’s modification of Black’s nuclear grading system (25), LVI, *in situ* component, tumor size, pathological tumoral (pT) and pathological nodal (pN) stages, and ER, PR and HER-2 status] was investigated for each group. Subsequent to quantitative comparison, four different Ki-67 cut-off values (≥10, ≥15, ≥20 and ≥25%) were defined. In addition, multivariate analysis was performed to identify any correlations between the Ki-67 cut-off values, the largest number of poor prognostic factors and the disease-free survival (DFS) rate.

### Statistical analysis

Statistical analyses were performed using SPSS 17.0 for Windows software (SPSS Inc., Chicago, IL, USA). The assessment of the association between Ki-67 expression and other prognostic factors was performed using χ^2^ tests (Pearson χ^2^, continuity correction and Fisher’s exact tests). In order to identify the independent prognostic factors associated with Ki-67 expression, a multivariate analysis using a logistic regression model was performed. The DFS rates were calculated using the Kaplan-Meier method. The differences in the DFS rates were evaluated using the log-rank test. DFS was defined as the time from diagnosis to the first locoregional tumor recurrence. The overall survival (OS) time was calculated as the length of time from BC diagnosis, until mortality from any cause. All P-values were two-sided, and P<0.05 was used to indicate a statistically significant difference.

## Results

### Patient characteristics

The patient characteristics are summarized in Table II. The median age was 49 years (range, 23–87 years), with 24.5% of the patients younger than 40 years old. The median Ki-67 level was 20% (range, 1–95%), and the majority of patients (80%) presented with IDC. Tumor multifocality/multicentricity, LVI and an *in situ* component were present in 17, 45 and 72% of cases, respectively. The ER, PR and HER-2 positivity rates were 81, 67 and 21%, respectively. In total, 53% of the patients presented with modified Scarff-Bloom-Richardson grade III tumors, and 61% exhibited Ki-67 levels of ≥15%. The pathological stage III rate was 21%, and the median tumor size was 20 mm. The median Ki-67 values and tumor sizes differed according to the distinct molecular subtypes of BC (Table I). The values were smallest in the luminal A group, and largest in the HER-2-enriched group.

### Qualitative and quantitative comparisons

**Figure 1:**
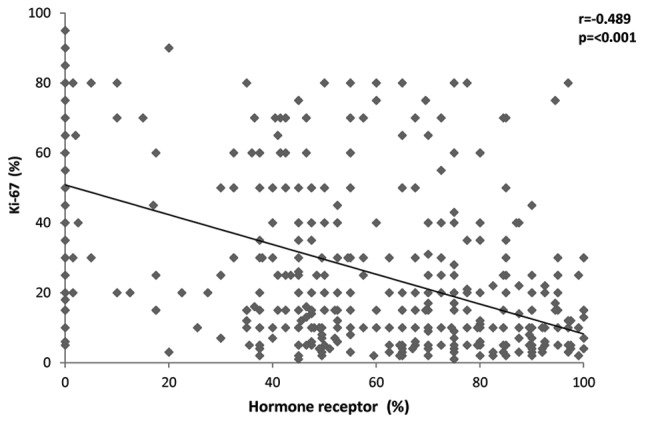
Regression analysis revealing the association between the hormone receptor positivity ratios and Ki-67 expression (n=462).

**Figure 2:**
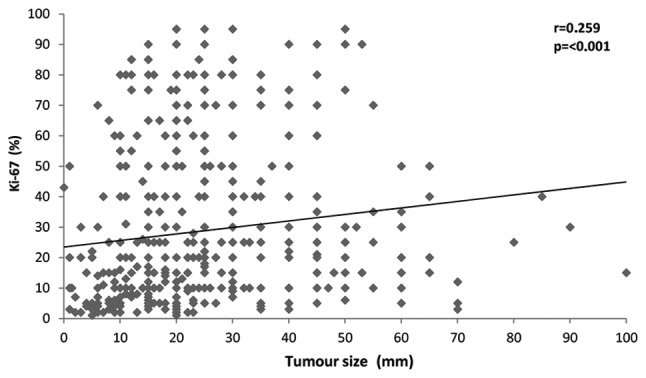
Regression analysis revealing the association between Ki67 expression and tumor size (n=462).

Subsequent to quantitative comparisons, a young age (≤40 years old; P<0.001), histological type (IDC; P=0.001), pT stage (pTII–III; P<0.001), a high grade (HG/NG III; P<0.001), LVI (P=0.001), ER/PR negativity (P<0.001) and HER-2 overexpression (P<0.001) were identified as demographical and pathological parameters associated with Ki-67 expression. The qualitative comparisons revealed a negative correlation between Ki-67 expression and the hormone receptor positivity ratio (r=−0.489; P<0.001), and a positive correlation between Ki-67 expression and tumor size (r=0.259; P<0.001) (Table III; Figs. 1 and 2).

### Multivariate analysis (logistic regression analysis)

The multiple logistic regression analysis revealed that a Ki-67 cut-off value of ≥15% was associated with the largest number of prognostic factors (≤40 years old, IDC histology, high HG III, ER negativity and HER-2 positivity) among the four selected cut-off values (≥10, ≥15, ≥20 and ≥25%) (Table IV).

### DFS

The median follow-up time was 25 months (range, 17–41 months). In total, locoregional and systemic tumor recurrence was observed in two and five patients, respectively. The Kaplan-Meier analysis revealed that the three-year DFS and OS rates were 97 and 100%, respectively. The univariate analysis revealed that ER negativity, a high HG (III), LVI and high Ki-67 expression (≥15%) were negatively associated with the DFS rate (Table V). Among the four Ki-67 cut-off groups in the luminal A and B1 molecular subtypes, a Ki-67 level of ≥15% was identified as the only statistically significant value. The DFS rate was higher in patients with the luminal A subtype compared with the luminal B1 subtype (100 vs. 93.6%; P=0.036; Table VI).

## Discussion

During the last decade, the overall incidence of BC has declined in the United States and other developed countries (26,27). However, the incidence of biologically aggressive BCs, such as the HR-negative, triple-negative and high-proliferative index subtypes has increased in low- to middle-income countries, and also in African-American populations in developed countries (19,27,28). Recent studies have identified a significant increase in HR-negative and high-grade BC subtypes within these populations (19,29–32). The HR-negative subtype, of which 50% of cases are also HER-2-negative, is a biologically aggressive form of BC, which is resistant to conventional cytotoxic chemotherapy, and is associated with a reduced survival rate compared with other known subtypes (33–36). These negative outcomes, and the high tumor proliferation rates observed, may be associated with a young age at the time of diagnosis, ethnicity and the different biological characteristics of tumor subtypes.

To the best of our knowledge, the present study is the first to assess the tumor proliferation status in a large cohort of Turkish females with BC. The retrospective analysis not only presented the Ki-67 cut-off value, but also investigated the association between the clinicopathological prognostic factors and the Ki-67 cut-off level. In total, 24.5% of the patients were <40 years old, and the median and optimal cut-off values of the Ki-67 level were 20 and 15%, respectively. Furthermore, 61% of patients exhibited a Ki-67 value of ≥15%, a level which represented a high-risk population.

The present study aimed to identify prognostic factors that were associated with Ki-67 expression. A younger age (≤40 years old), an IDC tumor type, HG/NG III, LVI, HR-negativity, HER-2 positivity and pT stage (tumor size) were revealed as poor prognostic factors associated with high expression levels of Ki-67. By contrast, the Ki-67 index was negatively correlated with HR positivity, and positively correlated with an increasing tumor size (P<0.001).

The majority of previous studies have identified the same poor prognostic factors associated with high levels of Ki-67. In particular, a higher tumor grade was revealed to be the most significant poor prognostic parameter (12,37–40,42). In a previous study, the reference cut-off value for Ki-67 expression was set at 15%, similar to the current literature (1,8,13), and the study identified a significant correlation between the tumor grading and the Ki-67 level (37). In the present study, the HG was revealed as an independent prognostic factor for each cut-off value group, and was correlated with a high level of Ki-67 expression. Previous studies demonstrated that the ER status was inversely associated with the Ki-67 index, and that low-proliferating tumors exhibited higher ER positivity rates (12,28,37–41), but that HER-2 expression rates were positively correlated with higher Ki-67 levels (28,37,38). In regard to tumor size, certain studies (12,30,38,42,43), including the present study, have identified a positive association with the Ki-67 index, however, others have not (37,40,41). A higher nodal status, which has been recognized as a significant prognostic factor for BC, has been associated with a higher Ki-67 index in previous studies (37,38,43), but not in the current study.

In recent decades, Ki-67 has been investigated as a potential immunohistochemical marker of proliferating cells, and an increasing amount of evidence now exists to support the use of Ki-67 as a clinical indicator of early BC (13). Despite the published studies that have analyzed the prognostic role of Ki-67 in BC, uncertainty remains concerning the assessment of Ki-67, partly due to the fact that the majority of the studies were retrospective (12,15). As no clear evidence exists regarding the methodology of how to interpret and score Ki-67 levels, or a definition of set Ki-67 cut-off values, the routine use of Ki-67 is not advocated. In 2011, Dowsett *et al* (21) published a set of recommendations for the worldwide standardization assessment of Ki-67 in BC. However, the methods used to evaluate the Ki-67 levels were highly variable across the laboratories, and were difficult to compare with the results of published studies (45). In the literature, the cut-off values were chosen or defined by investigators, and the threshold definitions of Ki-67 were accepted as mean or median values, or as an established arbitrary value (13). Cheang *et al* (45) identified that the ideal Ki-67 index cut-off value, to distinguish luminal A and B molecular subtypes, was 14%. This value was then recommended for clinical use in the 2011 St. Gallen International Expert Consensus on the Primary Therapy of Early Breast Cancer (1). The study by Cheang *et al* (45) presented two issues: i) Tissue microarrays, which were unable to reveal tumor heterogeneity as objectively as whole tissue sections, were used; and ii) the methodology used for Ki-67 counting was not clearly described. In two different meta-analyses, the cut-off values for the Ki-67 index varied from 0–34% (8,15). Despite the accepted 2011 St. Gallen Ki-67 cut-off value of 14% to differentiate between the luminal subtypes (1), the 2013 St. Gallen consensus identified a novel cut-off value of 20% (17). Randomized prospective studies may be designed to confirm the optimum Ki-67 cut-off value of 20%.

A recent study by Aleskandarany *et al* (43) evaluated the prognostic significance of Ki-67 within BC molecular subtypes. The study concluded that the Ki-67 index was able to distinguish between the luminal subgroups of patients with BC and different clinical outcomes. However, due to high proliferative activity, the Ki-67 index exhibited limited ability in stratifying HER-2-enriched and TNBC subtypes. In the present study, the median Ki-67 levels were 10, 30, 40 and 75% in luminal A and B, HER-2-positive and TNBC molecular subtypes, respectively (Table I). In accordance with previous studies (28,37,38,42), the levels of Ki-67 were significantly higher in patients with HER-2-positive and TNBC subtypes.

To determine the ideal Ki-67 cut-off value, and reveal any associations with poor prognostic factors and DFS/OS rates, the Ki-67 values were categorized into five groups (<10, 10–14, 15–19, 20–24 and ≥25%). In total, five independent poor prognostic factors (age of ≤40 years, IDC histological subtype, ER negativity, HG III and HER-2 positivity; Table IV) were identified to be associated with Ki-67 values of ≥15%. A Ki-67 index of >25% generally reflects an aggressive type of BC, and a systemic treatment decision is not challenging in highly proliferative tumors. In the present study, a patient age of ≤40 years, ER negativity and HG III were revealed to be associated with Ki-67 values of ≥25%.

TNBC is a heterogeneous disease, and exhibits different prognostic subgroups. High Ki-67 levels are often observed in cases of TNBC. Munzone *et al* (42) analyzed 496 node-negative TNBC patients, with a mean age of 52 years and a median Ki-67 level of 48% (range, 4–95%). The study revealed that the Ki-67 index increased with decreasing age and increasing tumor size and grade. Furthermore, the Ki-67 level was significantly higher in the ductal TNBC cases, compared with the other histological types. Nishimura *et al* (38) analyzed 2,638 BC patients, with a mean age, tumor diameter and Ki-67 value of 52.2 years, 2.2 cm and 20%, respectively. The majority of the cases were IDC, with a median Ki-67 index of 22%. The characteristics between this Japanese population and the Turkish females in the present study were quite similar. If the Japanese and Turkish BC patients were compared according to molecular subtypes (luminal A and B, HER-2-enriched and TNBC) the median Ki-67 values were identified as 17, 29, 40 and 50% in the Japanese series, and 10, 30, 40 and 75% in the present study, respectively. A higher Ki-67 index (>20%) was significantly correlated with a higher tumor grade and lower DFS and OS rates in the Japanese study.

The limitation of the present study was the short follow-up time (median, 25 months), and the small number of tumor recurrences (two loco-regional and five systemic) that were observed within this period of time. Therefore, the study mainly focused on the association between the prognostic parameters and the Ki-67 index, rather than the prognostic effect of the Ki-67 index on DFS and OS. The association between the poor prognostic factors and the high Ki-67 index was revealed in the study. The Kaplan-Meier estimates for the three-year DFS and OS rates were 97 and 100%, respectively. A Ki-67 value of ≥15% was revealed to be significant in its ability to distinguish between the HER-2-negative luminal A and B1 tumor subtypes, with a higher DFS rate in patients with the luminal A subtype compared with the luminal B1 subtype (P=0.036).

According to the St. Gallen 2013 consensus (17), a distinction between HER-2-negative luminal A and luminal B1 tumors can be made by ER, PR and Ki-67 expression levels, and can be determined only by laboratories with quality assurance programs. The strength of the present study was that all the pathological analyses were performed by a single, experienced breast pathologist in one laboratory. Previous, extended follow-up studies have included patients who had not received chemotherapy, or patients who had received a cyclophosphamide, methotrexate and fluorouracil regimen (40,42). The present, single-center study identified that for BC patients, a Ki-67 cut-off value of 15% was valuable for the distinction between molecular subtypes. This cut-off value was detected in patients treated with current systemic and irradiation modalities, and who were monitored for up to 41 months.

In conclusion, the optimal Ki-67 cut-off value for BC patients was identified as 15% for the distinction between different luminal subtypes. The high Ki-67 proliferation index was revealed to be significantly correlated with a young age, high tumor grade, IDC type, ER negativity and HER-2 positivity. An extended follow-up time is required in order to demonstrate the efficacy of the current treatment modalities for patients with different molecular subtypes and different Ki-67 cut-off values. The significance of the Ki-67 index has been investigated over the last decade, and may become a standard prognostic factor for future clinical use.

## Figures and Tables

**Table I:** Median Ki-67 levels according to molecular classification and histological type.

Tumor characteristics	n	%	Median Ki-67, %	Median tumor size, mm
Molecular subtype				
Luminal A/B1 (ER/PR^+^HER^−^)	313	67.7	10	20.0
Luminal B2 (ER/PR^+^HER^+^)	66	14.3	30	20.0
Triple negative (ER^−^PR^−^HER2^−^)	50	10.8	75	24.5
HER-2-enriched (ER^−^PR^−^HER2^+^)	33	7.1	40	28.0
Total	462	100.0	20	20.0
Histological type				
Invasive ductal cancer	368	79.7	20	
Other	94	20.3	10	

HER, human epidermal growth factor receptor; ER, estrogen receptor; PR, progesterone receptor.

**Table II:** Patients characteristics (n=462).

Patient characteristics	n	%	Median	Min-max
Age, years	462	-	49	23–87
≤40	113	24.5		
>40	349	75.5		
Tumor type				
IDC	368	79.7		
ILC	20	4.3		
Other	74	16.0		
Focality				
Unifocal	384	83.1		
Multifocal/multicentric	78	16.9		
Histological grade				
I–II	216	46.8		
III	246	53.2		
Nuclear grade				
I–II	173	37.4		
III	289	62.6		
Lymphovascular invasion				
Present	209	45.2		
Absent	253	54.8		
DCIS				
Present	332	71.9		
Absent	130	28.1		
Tumor size, mm	462	-	20	1–110
pT stage				
I	247	53.5		
II–IV	215	46.5		
pN stage				
0	240	51.9		
I–III	222	48.1		
pStage				
I	166	35.9		
II	198	42.9		
III	98	21.2		
ER				
Positive	376	81.4		
Negative	86	18.6		
PR				
Positive	309	66.9		
Negative	153	33.1		
HER-2 expression				
Positive	99	21.4		
Negative	363	78.6		
Ki-67 expression, %	462	-	20	1–95
<15	181	39.2		
≥15	281	60.8		

ER, estrogen receptor; PR, progesterone receptor; HER-2, human epidermal growth factor receptor-2; IDC, invasive ductal carcinoma; pT, pathological tumor; pN, pathological nodal.

**Table III:** Prognostic factors associated with Ki-67 expression (n=462).

	Ki-67 expression, %					
		
Prognostic factor	<10, n (%)	10–14, n (%)	15-<20, n (%)	20–24, n (%)	≥25, n (%)	P-value
Age, years						<0.001
>40	86 (84.3)	69 (87.3)	29 (76.3)	35 (83.3)	130 (64.7)	
≤40	16 (15.7)	10 (12.7)	9 (23.7)	7 (16.7)	71 (35.3)	a-bcde*; ab-cde***
Tumor type						0.001
IDC	69 (67.6)	58 (73.4)	33 (86.8)	34 (81.0)	174 (86.6)	
Other	33 (32.4)	21 (26.6)	5 (13.2)	8 (19.0)	27 (13.4)	ab-cd*; a-bcde**; ab-cde***
pT stage						<0.001
I	76 (74.5)	41 (51.9)	23 (60.5)	20 (47.6)	87 (43.3)	a-b**
II–III	26 (25.5)	38 (48.1)	15 (39.5)	22 (52.4)	114 (56.7)	a-bcde***; ab-cde***
pN stage						0.091
0	64 (62.7)	43 (54.4)	16 (42.1)	19 (45.2)	98 (48.8)	
I–III	38 (37.3)	36 (45.6)	22 (57.9)	23 (54.8)	103 (51.2)	ab-cd*; a-bcde*; ab-cde*
pStage						0.004
I	55 (53.9)	30 (38.0)	13 (34.2)	12 (28.6)	56 (27.9)	a-b*
II	35 (34.3)	30 (38.0)	17 (44.7)	20 (47.6)	96 (47.8)	a-bcde***; ab-cde***
III	12 (11.8)	19 (24.1)	8 (21.1)	10 (23.8)	49 (24.4)	
Focality						0.951
Unifocal	84 (82.4)	65 (82.3)	33 (86.8)	36 (85.7)	166 (82.6)	
Multifocal/multicentric	18 (17.6)	14 (17.7)	5 (13.2)	6 (14.3)	35 (17.4)	
HG						<0.001
I–II	87 (85.3)	56 (70.9)	21 (55.3)	19 (45.2)	33 (16.4)	a-b*; ab-cd***
III	15 (14.7)	23 (29.1)	17 (44.7)	23 (54.8)	168 (83.6)	a-bcde***; ab-cde***
NG						<0.001
I–II	70 (68.6)	44 (55.7)	12 (31.6)	17 (40.5)	30 (14.9)	b-c*; ab-cd***
III	32 (31.4)	35 (44.3)	26 (68.4)	25 (59.5)	171 (85.1)	a-bcde***; ab-cde***
LVI						0.001
Present	30 (29.4)	31 (39.2)	23 (60.5)	21 (50.0)	104 (51.7)	b-c*; ab-cd**
Absent	72 (70.6)	48 (60.8)	15 (39.5)	21 (50.0)	97 (48.3)	a-bcde***; ab-cde***
EIC						0.530
Present	67 (65.7)	59 (74.7)	27 (71.1)	29 (69.0)	150 (74.6)	
Absent	35 (34.3)	20 (25.3)	11 (28.9)	13 (31.0)	51 (25.4)	
ER						<0.001
Positive	99 (97.1)	76 (96.2)	36 (94.7)	39 (92.9)	126 (62.7)	
Negative	3 (2.9)	3 (3.8)	2 (5.3)	3 (7.1)	75 (37.3)	a-bcde***; ab-cde***
PR						<0.001
Positive	84 (82.4)	65 (82.3)	31 (81.6)	32 (76.2)	97 (48.3)	
Negative	18 (17.6)	14 (17.7)	7 (18.4)	10 (23.8)	104 (51.7)	a-bcde***; ab-cde***
HER-2						<0.001
Negative	102 (100.0)	69 (87.3)	29 (76.3)	28 (66.7)	135 (67.2)	a-b***; ab-cd***
Positive	0 (0.0)	10 (12.7)	9 (23.7)	14 (33.3)	66 (32.8)	a-bcde***; ab-cde***

pT, pathological tumor; pN, pathological nodal; HG, histological grade; NG, nuclear grade; LVI, lymphovascular invasion; EIC, extensive intraductal component; ER, estrogen receptor; PR, progesterone receptor; HER-2, human epidermal growth factor receptor-2. Letters are representative of each column: a, <10%; b, 10–14%; c, 15–20%; d, 20–24%; and e, ≥25%.

* P<0.05;

** P<0.01; and

*** P<0.001. (Pearson χ^2^, continuity correction and Fisher’s exact tests).

**Table IV:** Independent prognostic factors associated with Ki-67 expression (n=462).

A, ≥10% Ki-67						

Factors	B	S.E.	Wald	df	P-value	Exp(B)
IDC histology	0.747	0.290	6.612	1	0.010	2.110
Large tumor size (20 mm)	1.020	0.276	13.615	1	0.000	2.772
HG III	2.154	0.306	49.675	1	0.000	8.623
Constant value	1.472	0.172	73.421	1	0.000	4.356

B, ≥15% Ki-67						

Factors	B	S.E.	Wald	df	P-value	Exp(B)

Age (≤40 years)	0.718	0.290	6.105	1	0.013	2.049
Histologic type (IDC)	0.657	0.287	5.241	1	0.022	1.929
ER negativity	1.423	0.473	9.068	1	0.003	4.150
HG III	1.822	0.247	54.588	1	0.000	6.185
HER-2 positivity	1.323	0.384	11.878	1	0.001	3.756
Constant value	1.506	0.291	26.705	1	0.000	4.509

C, ≥20% Ki-67						

Factors	B	S.E.	Wald	df	P-value	Exp(B)

Age (≤40 years)	0.717	0.274	6.864	1	0.009	2.049
ER negativity	1.606	0.412	15.189	1	0.000	4.980
HG III	1.885	0.237	63.370	1	0.000	6.587
HER-2 positivity	0.923	0.315	8.592	1	0.003	2.516
Constant value	1.119	0.237	22.317	1	0.000	3.061

D, ≥25% Ki-67						

Factors	B	S.E.	Wald	df	P-value	Exp(B)

Age (≤40 years)	1.007	0.271	13.761	1	0.000	2.736
ER negativity	1.928	0.368	27.424	1	0.000	6.877
HG III	2.060	0.247	69.408	1	0.000	7.847
Constant value	0.469	0.204	5.278	1	0.022	1.599

Multivariate logistic regression model. IDC, invasive ductal carcinoma; HG, histological grade; ER, estrogen receptor; HER-2, human epidermal growth factor receptor-2; S.E., standard error; df, degrees of freedom.

**Table V:** Factors associated with DFS.

Factors	DFS, %	χ^2^	P-value
All cohorts	97.0		
ER		6.935	0.008^a^
Positive	98.0		
Negative	93.0		
Histological grade		6.745	0.009^a^
I–II	100.0		
III	94.1		
LVI		3.998	0.046^a^
Negative	99.4		
Positive	94.6		
Ki-67, %			
<10	100.0	2.461	0.117
≥10	96.0		
Ki-67, %		5.525	0.019^b^
<15	100.0		
≥15	94.6		
Ki-67, %		1.683	0.194
<20	98.2		
≥20	95.7		
Ki-67, %		3.297	0.069
<25	98.4		
≥25	94.8		

DFS, disease-free survival; ER, estrogen receptor; LVI, lymphovascular invasion.

a P<0.01;

b P<0.05.

χ^2^ was calculated by log-rank test.

**Table VI:** DFS according to the different molecular subtypes (n=462).

Molecular classification	Ki-67, %	DFS, %	χ^2^	P-value
Luminal-A	<10	100.0	1.980	0.159
Luminal-B1 [HER-2(−)]	≥10	95.7		
Luminal-A	<15	100.0	4.386	0.036^a^
Luminal-B1 [HER-2(−)]	≥15	93.6		
Luminal-A	<20	97.8	0.013	0.909
Luminal-B1 [HER-2(−)]	≥20	96.3		
Luminal-A	<25	98.0	0.152	0.697
Luminal-B1 [HER-2(−)]	≥25	95.5		
Luminal-B2 [HER-2(+)]	-	100.0	3.675	0.159
Triple(−)	-	91.2		
HER-2-enriched	-	93.2		

DFS, disease-free survival; HER-2, human epidermal growth factor-2.

a P<0.05.

χ^2^ was calculated by log-rank test.
